# Liking for Sweet Taste, Sweet Food Intakes, and Sugar Intakes

**DOI:** 10.3390/nu16213672

**Published:** 2024-10-29

**Authors:** Katherine M. Appleton

**Affiliations:** Department of Psychology, Faculty of Science and Technology, Bournemouth University, Poole BH12 5BB, UK; k.appleton@bournemouth.ac.uk; Tel.: +44-(0)1202-965985

**Keywords:** sweet, sugar, sucrose, preferences, liking, consumption

## Abstract

Background/Objectives: Sweet taste preferences are currently targeted to aid with reducing free sugar intakes, but associations between sweet taste liking, sweet food intakes, and sugar intakes are not well established. Methods: UK consumers (n = 179) who were consuming >5% of total energy intakes from free sugars provided several laboratory measures of sweet taste liking, laboratory test meal measures of sweet food choice and sugar intakes, and 3-day food diary measures of free-living free sugar and total sugar intakes. Liking measures included liking for a 1 M sucrose solution, and pleasantness, desire to eat, and sweet taste intensity ratings for seven foods of a range of sweet taste intensities in a taste test. Results: Wide individual differences in sweet taste liking, in liking for a high sweet taste intensity, and in the relationships between sweet taste intensity and sweet taste liking were found. The majority of participants confirmed high liking and increasing liking for increasingly sweet tastes, but differing patterns of responses were also found. Higher liking for sweet foods was associated with increased sweet food selection and consumption at the test meal, and to some degree with free sugar and total sugar consumption in this restricted scenario. However, we found no associations between sweet taste liking, regardless of measure, and either free-living free sugar or total sugar intakes. Conclusions: These findings cast doubt on assumptions that sweet taste preferences are high for all and that these high sweet taste preferences drive high free sugar intakes.

## 1. Introduction

High intakes of free sugars, defined as ‘*monosaccharides and disaccharides added to foods and beverages by the manufacturer, cook or consumer, and sugars naturally present in honey, syrups, fruit juices and fruit juice concentrates*’ by the World Health Organization (WHO) [1] (p. 4), are associated with increased risk from dental caries, excess energy intake, overweightness, and obesity [1]. As a result, the WHO currently recommends ‘*In both adults and children, the intake of free sugars should be reduced to less than 10% of total energy intake*’ [1] (p. 4), and ‘*a reduction to less than 5% of total energy intake would provide additional health benefits*’ [1] (p. 4).

To aid with this reduced consumption of sugars, some public health agencies further recommend the reduced consumption of sweet foods, regardless of the source of the sweet taste, in an attempt to reduce sweet taste preferences [2,3]. Whether sweet taste preferences are impacted by sweet food consumption remains a topic of current debate [4,5], but implicit in this recommendation are also the suggestions that sugar consumption is a result of sweet taste preferences and that sweet taste preferences are naturally high.

Innate preferences or an innate liking for sweet taste is well known [6], but individual differences in degree of liking and most preferred concentration or sweet taste intensity are also found [7,8,9,10,11,12]. Differences in degree of liking are found in studies where participants report the extent to which they like one or several sweet-tasting stimuli [7,8], while differences in most preferred concentration are reported where participants are asked to rate, rank, or choose from multiple stimuli with varying concentrations of sweet taste [8,9,10,11,12]. Where multiple concentrations are involved, sweet taste ‘likers’ can also be defined as individuals with an increasing liking for increasingly intense sweet tastes, while for sweet taste ‘dislikers’, a decreasing intensity is preferred. For others, the optimal sweet taste intensity lies somewhere in the middle, at a range of values at the midpoint of an inverted U-shaped function, or there is no distinguishable optimum. Importantly, the term ‘sweet liker’ refers to liking a high *concentration* of sweet taste, rather than a high liking for sweet taste, and this distinction between liking for a taste and liking for a taste intensity, while likely related, is often confused. Thus, while sweet taste liking may be almost universal, for some individuals, sweet foods are highly liked, for others, sweet foods are less liked; and for some individuals, highly sweet foods are highly liked, while for others, less sweet foods are more liked.

It is plausible, furthermore, that liking for sweet taste will be associated with sweet food intakes. Associations are demonstrated where sweet taste liking and sweet food consumption are explicitly measured [7], but associations between liking for a highly sweet taste and sweet food intakes may depend more on the sweet taste intensity of the foods that are available [11,12].

Liking for a sweet taste and liking for a high sweet taste intensity may also not necessarily map sugar intakes. Low-calorie sweeteners (LCS) provide the pleasure of sweet taste in the absence of sugar or for a much reduced use of sugars [13]; thus, a high sweet taste liking can be satisfied without the consumption of sugars. Furthermore, not all high-sugar foods are considered sweet or highly sweet; some processed foods that are high in sugars are foods that the average consumer would consider savoury. Pasta sauces and discretionary sauces, such as ketchup, canned vegetables, premade soups, and take-aways, can be very high in sugar [14]. Considering this mismatch between sweet taste and sugar content, sweet taste liking and liking for a high sweet taste intensity may not necessarily be associated with sugar intakes.

Sugar consumption, furthermore, is likely associated with a lot more than preference for a sweet taste. Attitudes towards sugars, sweeteners, and sweet-tasting foods, and other socio-cognitive determinants, such as self-efficacy, have been associated with sugar intakes and the intake of sugar-sweetened foods [15,16,17]. As part of a complete dietary pattern, sugar intakes will also likely be affected by attitudes that impact wider consumption behaviours, such as concerns over health or body weight [16,17].

Other studies have sought to investigate associations between sweet food preferences and sugar intakes. In a recent systematic review [18], studies that assessed both liking for sweet taste and liking for a high sweet taste intensity report associations with various aspects of dietary consumption, including sugar intakes [10,11,12]. Not all studies, however, report these associations [19,20], and few studies tease apart liking for the taste and liking for a high sweet taste intensity, or consider attitudinal drivers of food intake.

This analysis sought to explore the associations between sweet taste liking, liking for a high sweet taste intensity, sweet food intakes, and sugar intakes in a sample of UK consumers. It was hypothesized that:

- a range of values for liking for sweet taste and liking for a high sweet taste intensity would be found;
- a range of relationships between liking for sweet taste, sweet taste intensity, and liking for a high sweet taste intensity would be found;
- liking for sweet taste and liking for a high sweet taste intensity would be associated with sweet food intakes;
- liking for sweet taste and liking for a high sweet taste intensity would be associated with sugar intakes.

## 2. Materials and Methods

Data were gained from a randomised controlled trial where 242 adults from the UK consuming >5% total energy intake (TEI) from free sugars were randomised to receive different dietary recommendations to reduce their free sugar intakes (see [21] for the study protocol). At study start, all participants completed three-day food diaries to provide a measure of their general diet, and 179 (74%) of these participants also attended a laboratory test session. The analyses presented here are based on the food diary and laboratory data from these 179 participants. Food diaries provided measures of free-living sugar intakes. The laboratory test session provided measures of sweet taste liking, sweet food intakes, and additional measures of sugar intakes. This is a secondary analysis of data that were collected as part of the trial [21].

### 2.1. Participants

Participants for the trial were healthy adult volunteers who were consuming >5% TEI from free sugars, as assessed from food diaries completed over three non-consecutive days, as detailed below; had no pre-existing medical conditions affecting their willingness or abilities to reduce their free sugar intakes, e.g., diabetes mellitus; had no pre-existing medical conditions affecting swallowing ability, taste, and/or smell perception; and were undertaking no behaviours that might affect their willingness or ability to reduce free sugar intakes or swallowing ability, taste, and/or smell perception, e.g., dieting, smoking.

### 2.2. Food Diaries

Diaries were completed for three non-consecutive days, comprised of two weekdays and one weekend day [22]. Multiple food diaries, such as these, are considered to be a good measure of short-term dietary intake that are suitable for assessing short-term changes to intakes, as was required for the trial [22,23,24]. To retain research integrity while reducing research burden, participants were asked to record all foods and beverages consumed across each day, using electronic recording [24,25] via Nutritics software (research edition, version 5) and the Libro recording app [26]. Nutritics software is supported by an extensive food database, where food composition data are repeatedly internally validated [26]. Training was given to participants prior to diary completion by a researcher trained in food diary methodology, and accuracy was checked on submission [22]. Where diaries were not sufficiently detailed, or suspected as incomplete by this researcher, potential study participants were excluded from the study prior to the study start.

Completed diaries were subsequently analysed as an average of the three days. Total sugar content and free sugar content for all recorded foods were calculated using manufacturer’s information. Where sugar or free sugar information was unavailable, consumed foods were replaced in full with the nearest alternative food for which all information was available. Free sugar intakes and total sugar intakes were then calculated as a percentage of TEI to provide measures that are named ‘diary %FS’ and ‘diary %TS’, respectively.

All foods and beverages consumed were also categorised as ‘high-sugar’, ‘medium-sugar’, or ‘low/no-sugar’, based on the criteria used for the UK traffic light food labelling system [27], where high-sugar foods have >22.5 g sugar/100 g, medium-sugar foods have 5–22.5 g sugar/100 g, and low/no-sugar foods have <5 g sugar/100 g. Grams of each food type consumed/day were then summed and divided by the total weight of foods and beverages consumed/day, to give grams of high- and medium-sugar foods consumed as a percentage of all foods consumed. These measures are named ‘diary %HS’ and ‘diary %MS’, respectively.

### 2.3. Laboratory Test Session

The laboratory test session provided measures of sweet taste liking, liking for a high sweet taste intensity, sweet taste intensity perceptions, sweet food consumption, and additional measures of sugar intake.

Liking for a high sweet taste intensity was assessed first using a 1 M aqueous sucrose solution. Ratings of a 1 M aqueous sucrose solution have recently been suggested as a parsimonious method to categorise individuals as ‘sweet likers’, those responding with ‘an inverted U-shaped function’, and ‘sweet dislikers’ in a single test session [28,29]. Participants consumed 10 mL samples of the solution and rated sweet taste intensity using a 100 mm pen and paper version of a general labelled magnitude scale, following training in this method, then rated their liking for the sweet solution using 100 mm visual analogue (VAS) scales [28,29,30,31]. While individuals were then categorised as ‘sweet likers’, ‘inverted-U’, and ‘sweet dislikers’ for the purposes of the trial [21], continuous scores for liking for the highly sweet solution were used for this analysis to increase sensitivity and avoid the possibility of effects as a result of categorisation [20].

Sweet taste liking and sweet taste intensity perceptions were then assessed using a taste test comprised of seven commercially available sweet and non-sweet foods commonly consumed in the UK. These foods were a Kellogg’s Crunchy Nut Cornflake, a Kellogg’s Fruit ‘n’ Fibre flake with raisin, a 5 g sample of strawberry jam, a 10 mL sample of orange juice (sweet foods), a Kellogg’s cornflake, a Kellogg’s bran flake, and a 5 g sample of smooth peanut butter (non-sweet foods). Food samples were provided to participants to be consumed in full in a pre-determined order. At the time of consumption, foods were rated on 100 mm VAS for pleasantness: ‘*How pleasant is this food right now? (not at all–extremely)*’; desire to eat: ‘*How strong is your desire to eat this food right now? (not at all–extremely)*’; and sweet taste intensity: ‘*How sweet is this food right now? (not at all–extremely)*’ [30,31]. Foods were interspersed with a mouthful of water and a bite of a cream cracker to cleanse the palate. This food-based taste test provided an ecological assessment of sweet taste liking and sweet taste intensity perceptions. Much sweet taste testing uses sucrose-sweetened aqueous solutions [18,30,31], yet individuals do not typically consume flavourless sucrose-sweetened aqueous solutions, and in realistic scenarios, sweet taste perception will be affected by other aspects of a food or beverage, such as flavour and texture [9,10,11,20,31]. The use of sweet and non-sweet foods allowed investigation over a range of sweet taste intensity values. Use of a pre-determined order ensured foods were consumed in a similar order at several time points (not included in these analyses). Multiple pre-determined orders were used across participants to avoid effects as a result of the order in which the foods were presented. The consumption of all taste test items in full ensured against effects in test meal consumption as a result of differences in intake during the taste test [32].

Ratings of pleasantness, desire to eat, and sweet taste intensity for all seven individual foods were retained as individual ratings, and ratings for the four sweet foods and the three non-sweet foods were also subsequently combined and averaged to provide measures of pleasantness, desire to eat, and sweet taste intensity for sweet foods and non-sweet foods, respectively.

Following the taste test, participants were provided with an ad-libitum buffet-style breakfast meal. This meal was composed of a range of sweet and non-sweet foods, suitable for breakfast in the UK. Non-sweet foods were bagels (plain), butter, cream cheese, peanut butter, cornflakes, and bran flakes. Sweet foods were bagels (cinnamon and raisin), strawberry jam, honey, Crunchy Nut Cornflakes, and Fruit ‘n’ Fibre. Milk, water, orange juice, caffeinated and decaffeinated coffee and tea were also provided, with optional sugar or -LCS. Full details of the foods are given in the Supplementary Materials, Table S1. Foods were provided in ad-libitum portions to ensure choice, and participants were free to consume as little or as much as they wished of any and all foods to allow a reflection of sweet and non-sweet food choice [33]. All foods were weighed individually prior to their provision to each participant, on an individual basis, and weighed again on completion of the breakfast to provide a weight of all individual foods and beverages consumed [33].

Sweet food consumption was subsequently calculated using weight from sweet foods consumed as a percentage of total weight consumed, and energy consumed from sweet foods as a percentage of total energy consumed. These measures are named ‘meal %weight sweet’ and ‘meal %energy sweet’, respectively. Two measures were used, because appropriate measures of sweet taste consumption within the diet have not yet been established [31], and use of these specific measures allowed the use of measures based on energy consumed [34,35] and allowed consideration of foods and beverages that can be highly sweet but that contribute very little energy to the diet, e.g., low-calorie-sweetened beverages. Manufacturer’s information was also used to convert amount consumed into sugars consumed, percent energy consumed in the meal from sugars, free sugars consumed, and percent energy consumed from free sugars. These variables are named ‘meal sugars’, ‘meal %energy sugars’, ‘meal free sugars’, and ‘meal %energy free sugars’, respectively.

### 2.4. Additional Measures

Some additional variables were also assessed as potential confounders in the relationships between sweet taste liking, sweet food intakes, and sugar intakes [10,15,16,17,19,30,31,34,35,36]. These were: demographic variables: gender (male/female), age (in years), and body mass index (BMI); attitudinal variables: attitudes towards sugars, sweeteners and sweet-tasting foods (SSSF), and attitudes towards eating behaviours; and appetite at the start of the test day. Attitudes towards SSSF were measured by questionnaire [17]. This questionnaire, based on earlier qualitative work [36], uses 48 questions to assess six factors of importance in the consumption of SSSF: Personal Impact (SSSF have an influence or impact on the individual) (10 items); Personal Management (the individual feels in control or able to manage their intakes of SSSF) (13 items); Apathy (apathy or nonchalance towards SSSF) (5 items); Negativity (unfavourable perceptions of SSSF) (7 items); Perceived Understanding (knowledge and awareness of SSSF) (8 items); and Perceived Nonautonomy (a lack of autonomy or control over SSSF consumption) (5 items). Attitudes towards eating behaviours were measured using the Three Factor Eating Questionnaire (TFEQ-R18 [37]). This questionnaire uses 18 items to assess three aspects of eating behaviour: Cognitive Restraint (the conscious restriction of food intake, usually for weight-related reasons) (6 items); Uncontrolled Eating (loss of control over eating, often resulting in over-consumption) (9 items); and Emotional Eating (eating in response to emotional cues) (3 items). Appetite at the start of the test day was assessed using 100 mm VAS ratings of hunger: ‘*How hungry are you? (not at all–extremely)*’; desire to eat: ‘*How strong is your desire to eat? (not at all strong–extremely strong)*’; fullness: ‘*How full are you? (not at all–extremely)*’; and prospective consumption: ‘*How much could you eat right now? (nothing at all–a large amount)*’.

### 2.5. Procedure

Following consent procedures, participants completed all eligibility assessments, including food diaries for three days. Participants who were eligible for the trial and consumed >5% TEI from free sugars in these diaries were then invited to attend a laboratory test session. Participants were required to attend the test session from 7 a.m.–11 a.m. following an overnight fast. On arrival, participants first reported their (fasting) appetite, then undertook assessments for the calculation of BMI, completed by a trained researcher. They then completed the solution taste test, the food-based taste test, and were then provided with the buffet-style meal. Laboratory measures were undertaken following an overnight fast to ensure against effects due to earlier consumption and taste experiences. Prior to arriving at the laboratory, participants completed all questionnaire measures. These were checked in advance by the researcher and any inconsistencies were checked with the participant during breakfast.

The trial was undertaken from April 2021 to December 2022 in Bournemouth, UK, with data for this analysis collected from April 2021 to September 2022. Following all procedures, as above, the following measures were available for all 179 participants:

- For sweet taste liking: ratings of liking for a 1 M aqueous sucrose solution and ratings of pleasantness and desire to eat for all seven foods, and for sweet and non-sweet foods in the taste test;
- For sweet taste intensity: ratings for sweet taste intensity for all seven foods, and for sweet and non-sweet foods in the taste test;
- For sweet food intakes: meal %weight sweet, meal %energy sweet;
- For sugar intakes: diary %FS, diary %TS, diary %HS, dairy %MS, meal sugars, meal %energy sugars; meal free sugars, meal %energy free sugars;
- Additional measures: gender; age; BMI; attitudes towards SSSF: SSSF questionnaire sub-scales: Personal Impact; Personal Management; Apathy; Negativity; Perceived Understanding; Perceived Nonautonomy; attitudes towards eating behaviours: TFEQ sub-scales: Cognitive Restraint; Uncontrolled Eating; Emotional Eating; Appetite measures: hunger; desire to eat; fullness; prospective consumption.

### 2.6. Analysis

All data were processed at the end of the trial in a single time period to ensure consistency. The sample for these analyses was then described based on demographic characteristics and all variables of interest.

To investigate the range of values for liking for sweet taste, mean pleasantness ratings and desire to eat ratings for all sweet foods and ratings for liking for the 1 M sucrose solution for the whole population were assessed.

To investigate associations between sweet taste liking and sweet taste intensity, first, correlations were run between ratings for sweet taste intensity and ratings for pleasantness and desire to eat for all seven foods in the taste test, per individual. Second, range in sweet taste liking based on sweet taste intensity was investigated per individual. Third, mean sweet taste intensity ratings for sweet foods and non-sweet foods were correlated with mean pleasantness and desire to eat ratings across the whole study sample to confirm any associations found on an individual basis. Fourth, correlations were run between mean pleasantness and mean desire to eat ratings for the sweet and non-sweet foods, liking for the 1 M sucrose solution, and the individual relationships between pleasantness, desire to eat, and sweet taste intensity ratings.

To investigate associations between liking for sweet taste, liking for a high sweet taste intensity, and sweet food intakes, correlations were run between mean pleasantness and desire to eat ratings for sweet foods, ratings of liking for the 1 M sucrose solution, and both measures of sweet food intake (meal %weight sweet and meal %energy sweet).

Regression analyses were then run where each of the sweet food intake outcomes was predicted by all sweet taste liking and intensity measures. Ratings for pleasantness and desire to eat were highly correlated (r = 0.858, *p* < 0.01), so only ratings for pleasantness were included. Age, gender, BMI, SSSF attitudes, eating attitudes, and hunger were also included in these models. Hunger and desire to eat were highly correlated (r = 0.848, *p* < 0.01), and both hunger and desire to eat were negatively correlated with fullness (r = −0.488, *p* < 0.01, r = −0.494, *p* < 0.01, respectively) and positively correlated with prospective consumption (r = 0.494, *p* < 0.01, r = 0.484, *p* < 0.01, respectively). To avoid concerns over mutli-co-linearity, only hunger was used in all models.

To investigate associations between liking for sweet taste, liking for a high sweet taste intensity, and sugar intakes, correlation and regression analyses were run as above for all eight measures of sugar intake (diary %FS, diary %TS, diary %HS, dairy %MS, meal sugars, meal %energy sugars, meal free sugars, and meal %energy free sugars). Models for sugar intakes from diaries were also repeated to include measures from the test meal to investigate the value of these. Meal %weight sweet and meal %energy sweet (r = 0.786, *p* < 0.01), meal sugar intakes and free sugar intakes (r = 0.933, *p* < 0.01)), and meal %energy sugar and %energy free sugars (r = 0.852, *p* < 0.01) were highly correlated, so only meal %weight sweet, meal sugars, and meal %energy sugars were included.

Analyses were conducted in SPSS, version 28.0. Significance was set at *p* < 0.05. Given the exploratory nature of the work, marginally significant results (*p* = 0.05 to *p* = 0.08) were also identified where these conferred with significant effects in a similar measure, to ensure no effects were missed. *p*-values are used to denote effects of interest considering the absence of any suggestion at present on the clinical significance of any measure of sweet taste liking or preference [31].

## 3. Results

### 3.1. Participants

Characteristics of the 179 participants involved in these analyses are given below, with full details as recorded for the trial also provided in the Supplementary Materials, Table S2. The sample consisted of 21 males and 158 females, with a mean (SD) age of 40.6 (13.7) years, ranging from 18 to 63 years, and a mean (SD) BMI of 27.8 (5.9) kg/m^2^, ranging from 18.6 to 46.1 kg/m^2^. Participants reported consuming a mean (SD) 1733 (507) kcal/day in the food diaries and consumed a mean (SD) 291 (259) kcal at the breakfast meal. Descriptive statistics (mean, SD, and range) for sweet taste liking, liking for a high sweet taste intensity, sweet food intakes, and sugar intakes are given in Table 1.

### 3.2. Liking for Sweet Taste and Liking for a High Sweet Taste Intensity

Pleasantness and desire to eat ratings for sweet foods ranged between 2 and 95 mm, and 0 and 92 mm, respectively, following a normal distribution. Considerable variation in liking for a high sweet taste intensity was also found, with values ranging from 0 to 99 mm, again following a normal distribution. Figures are presented in the Supplementary Materials, Figures S1–S3.

### 3.3. Sweet Taste Liking and Sweet Taste Intensity

**Figure 1:**
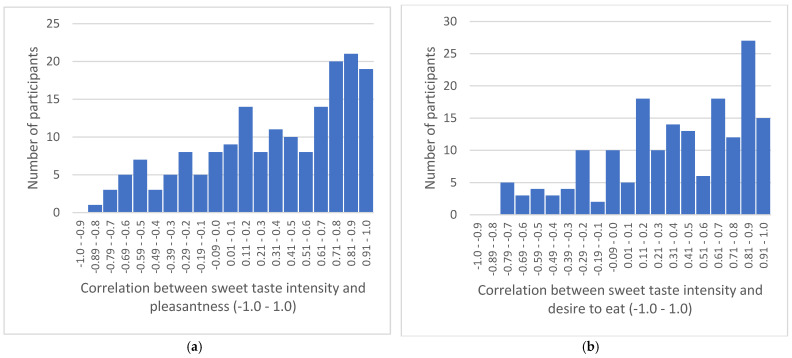
Histograms demonstrating the number of participants displaying each correlation between sweet taste intensity and pleasantness rating (**a**) and desire to eat rating (**b**) for the seven foods varying in sweet taste intensity in the taste test (n = 179).

Correlations between sweet taste intensity perception, pleasantness, and desire to eat ratings for foods with a range of concentrations of sweet taste per individual ranged from r = −0.87 to 1.00 and r = −0.79 to 1.00, respectively, and these are presented in the histograms in Figure 1. Some participants reported strong negative correlations between sweet taste intensity and sweet taste liking; thus, high sweet taste intensity is associated with low sweet taste liking, while some participants reported strong positive correlations, where high sweet taste intensity is associated with high sweet taste liking. Negative skew statistics (pleasantness −0.562; desire to eat −0.621) demonstrate that more individuals reported a positive association.

**Figure 2:**
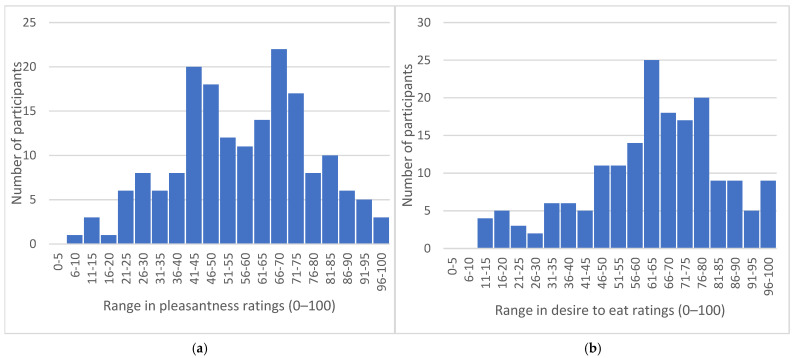
Number of participants reporting each range in pleasantness ratings (**a**) and desire to eat ratings (**b**) over the seven foods varying in sweet taste intensity in the taste test (n = 179).

Range in pleasantness and desire to eat ratings for all seven foods with differing concentrations of sweet taste intensity in the taste test, per individual, ran from 9 to 100 mm and 12 to 100 mm, respectively, as presented in the histograms, Figure 2. Some participants reported very limited differences in liking for foods with very different concentrations of sweet taste, while some participants reported very large differences.

**Figure 3:**
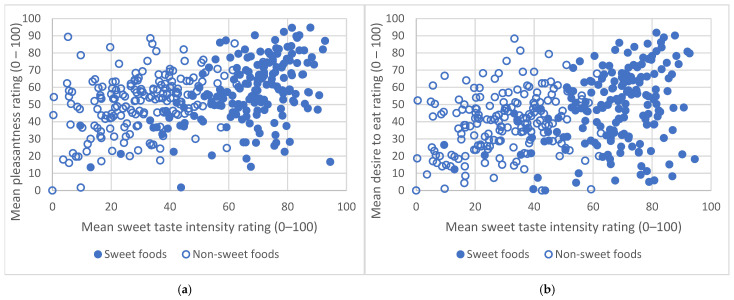
Scatterplots demonstrating the correlation between mean sweet taste intensity and mean pleasantness rating (**a**) and mean desire to eat rating (**b**) over the four sweet foods and the three non-sweet foods in the whole population (n = 179).

For the population as a whole, mean sweet taste intensity for the four sweet foods and the three non-sweet foods was positively correlated with mean pleasantness and mean desire to eat ratings (sweet foods pleasantness r = 0.452, *p* < 0.01, desire to eat r = 0.348, *p* < 0.01; non-sweet foods pleasantness r = 0.366, *p* < 0.01, desire to eat r = 0.259, *p* < 0.01); see Figure 3.

### 3.4. Sweet Taste Liking and Liking for a High Sweet Taste Intensity

Significant positive associations were found between liking for a high sweet taste intensity and mean pleasantness and mean desire to eat ratings for sweet foods (r = 0.299, *p* < 0.01, r = 0.314, *p* < 0.01, respectively), while no associations were found with mean pleasantness ratings for non-sweet foods (r = 0.022, *p* = 0.08) and marginal effects were found in mean ratings for desire to eat non-sweet foods (r = 0.148, *p* = 0.05). Regression analyses taking hunger into account revealed this effect in desire to eat to be related to hunger rather than high sweet taste intensity liking. Results from the regression analyses for each outcome are given in the Supplementary Materials, Table S3.

Mean pleasantness and mean desire to eat ratings for sweet foods and ratings of liking for a high sweet taste intensity were also positively correlated with individual relationships between sweet taste intensity and pleasantness (r = 0.298, *p* < 0.01 to r = 0.403, *p* < 0.01) and desire to eat all seven foods (r = 0.243, *p* < 0.01 to r = 0.443, *p* < 0.01).

### 3.5. Liking for Sweet Taste, Liking for a High Sweet Taste Intensity, and Sweet Food Intakes

Mean ratings for pleasantness and desire to eat sweet foods were positively corelated with %weight consumed from sweet foods (pleasantness r = 0.295, *p* < 0.01; desire to eat r = 0.333, *p* < 0.01) and %energy consumed from sweet foods at the breakfast meal (pleasantness r = 0.225, *p* < 0.01; desire to eat r = 0.295, *p* < 0.01).

Liking for a high sweet taste intensity was not correlated with the measures of sweet food intake, although a marginal effect was found for %weight consumed from sweet foods at the breakfast meal (meal %weight sweet r = 0.142, *p* = 0.06, meal %energy sweet r = 0.092, *p* = 0.22).

Results from the regression analyses for both measures of sweet food consumption are given in Table 2. Only %weight consumed from sweet foods was significantly predicted by the regression model. A greater %weight consumed from sweet foods was associated with greater pleasantness/desire to eat ratings for the sweet foods.

### 3.6. Liking for Sweet Taste, Liking for a High Sweet Taste Intensity, and Sugar Intakes

Mean pleasantness and desire to eat ratings for sweet foods were positively correlated with sugars consumed (pleasantness r = 0.187, *p* = 0.01; desire to eat r = 0.313, *p* < 0.01) and free sugars consumed at the breakfast meal (pleasantness r = 0.213, *p* < 0.01; desire to eat r = 0.326, *p* < 0.01), and desire to eat ratings for sweet foods were positively corelated with % energy consumed from free sugars (r = 0.180, *p* = 0.02). Associations with the other measures of sugar intake were not found (largest r = 0.127, *p* = 0.09).

Liking for a high sweet taste intensity was positively corelated with %FS, as calculated from food diaries (r = 0.185, *p* = 0.01), with free sugar intakes at breakfast (r = 0.155, *p* = 0.04), and marginally with sugar intake at the breakfast (r = 0.132, *p* = 0.08), but not with other measures of sugar intake (largest r = 0.067, *p* = 0.37).

Results from the regression analyses on four measures of sugar consumption are given in Table 3. Both diary measures, meal sugars, and meal free sugars were significantly predicted by the regression models. Greater diary %FS was associated with lower agreement with the SSSF factor Apathy and lower agreement with the TFEQ factor Cognitive Restraint. Greater diary %TS was associated with a higher age, and greater agreement with the SSSF factor Personal Management. Meal sugars and meal free sugars were associated with higher pleasantness/desire to eat ratings, lower sweet taste intensity ratings, and higher agreement with the SSSF factor Negativity.

Other measures of sugar consumption were not predicted by the regression models (%High-Sugar food consumption: R^2^ = 0.09, adjusted R^2^ = 0.00, F(17,178) = 0.95, *p* = 0.51; %Medium-Sugar food consumption: R^2^ = 0.12, adjusted R^2^ = 0.03, F(17,178) = 1.34, *p* = 0.18; meal %energy from sugars: R^2^ = 0.10, adjusted R^2^ = 0.01, F(17,178) = 1.04, *p* = 0.42; meal %energy from free sugars: R^2^ = 0.13, adjusted R^2^ = 0.04, F(17,178) = 1.38, *p* = 0.16).

## 4. Discussion

This analysis sought to explore the associations between sweet taste liking, liking for a high sweet taste intensity, sweet food intakes, and sugar intakes, in a sample of UK consumers consuming >5% TEI from free sugars. A number of interesting findings emerged.

First, there was a wide range in liking for sweet taste. Overall, in the population as a whole, sweet taste was liked, and higher concentrations of sweet taste were associated with higher liking, but some participants also provided low ratings for pleasantness and desire to eat for the sweet foods in the taste test, low ratings of liking for a high sweet taste intensity, and negative relationships between sweet taste intensity and sweet taste liking, or reported liking for the foods that was little affected by the intensity of sweet taste. The first two hypotheses, that a range of values for liking for sweet taste and liking for a high sweet taste intensity, and a range of relationships between liking for sweet taste, sweet taste intensity, and liking for a high sweet taste intensity would be found, were supported. These findings confirm the population-wide liking for sweet taste that is well known and demonstrate the individual differences that have also previously been reported [7,8,9,10,11,12].

The similarities between different methods, and the absence of similar associations for non-sweet foods, also suggest an underlying validity to these different measures [9,11,19,28,29,30]. Differences however, were also found, and these differences may be important; liking for a high sweet taste intensity and other measures of sweet taste liking were significantly, but not highly, correlated. Differences were also found between these differing measures when investigating associations with test meal intakes. Pleasantness and desire to eat ratings for the foods in the taste test were more highly associated with sweet food and sugar intakes in the test meal, while no associations were found with liking for a high sweet taste intensity or the individual relationships between sweet taste intensity and sweet taste liking. The hypothesis that liking for sweet taste would be associated with sweet food intakes is supported, while the hypothesis that liking for a high sweet taste intensity would be associated with sweet food intakes is not supported. It maybe unsurprising that liking for foods in a taste test predicts their subsequent intake, and this has been previously demonstrated [7]. Relationships between sweet food liking and sweet food intakes in a more general sense have also been reported [18], and the distinction demonstrated in the analyses presented here based on differing measures may help explain inconsistencies in earlier reports [18].

While associations are found, it is also worth adding that the clinical significance of these effects and the importance of effect size remains largely unknown [31]. Public health agencies claim a value to lowering sweet food preferences for limiting sweet food intakes [2,3], but at present there is very little evidence that sweet food preferences or sweet food intakes are associated with health outcomes [31,34,35,38,39,40].

The hypothesized associations between liking for sweet taste and sugar intakes were also found when sugar intakes were assessed in a laboratory test meal, while the hypothesized associations between liking for a high sweet taste intensity and sugar intakes were not found. The hypothesis that liking for sweet taste would be associated with sugar intakes is also not supported when considering free-living sugar intakes. These findings demonstrate clear differences, again, based on the measure used. Sugars consumed in the test meal were associated with sweet taste liking, although the effects are not strong and will be constrained by the laboratory situation [32,33]. There is some suggestion also that sugar intakes in the taste meal were negatively associated with perceived sweet taste intensity, an effect that has been reported previously [41,42], but the importance of any association between sweet taste intensity, sweet food intakes, and sugar intakes, at present, remains unclear [18].

Importantly, free sugar intakes and total sugar intakes from diaries were not associated with any of the measures of sweet food liking. These findings cast doubt on the suggested association between sweet taste liking and sugar intakes that is given in public health messaging [2,3]. A lack of association between sweet food preference measures and sugar intakes [9,11,18] has previously been attributed to the methods used to assess sweet food liking [18,19] and variation based on tastant, strength of tastant, and test scenario [10,11,12,20]. Where associations have been reported [12,18], not all studies demonstrate these [18], or studies demonstrate relationships that are limited and small in effect size [19,20], or more complex or nuanced [10,12]. It is possible, however, that a generalized liking or sweet taste preference is not sufficient for sugar consumption and that other aspects of a food item, such as flavour or texture [9,10,11,20,31], and other factors, such as attitudes, are also important for selection and consumption in the real world.

Free sugar intakes and total sugar intakes in this analysis were significantly associated with attitudes towards sugars, sweeteners, and sweet foods, and with attitudes towards wider eating behaviours. Higher free sugar intakes as a percentage of total energy intake were associated with less ‘apathy’ towards sugars, sweeteners, and sweet tasting foods, and lower cognitive restraint. Lower apathy towards sugars, sweeteners, and sweet foods may suggest an active choice in their consumption [17,36], but further work is clearly needed here. Associations with lower cognitive restraint are unsurprising, and most likely reflect the highly pleasing nature of sweet taste and the effort required to control or limit consumption [6,36]. Total sugar intakes were also associated with attitudes towards sugars, sweeteners, and sweet foods, both in the diaries and in the test meal. These associations suggest high sugar intakes where participants believe they can manage their intakes, as has previously been reported [17], and in those who have a negative perception of LCS, an effect that also may be unsurprising [36]. Total sugar intakes were also positively associated with participant age, and in the test meal, with hunger. Only one previous study investigating sweet taste liking, of which we are aware, also distinguishes between free sugar and total sugar intakes [11]. In this study, Holt et al. [11] found stronger associations between sweet taste liking and the consumption of refined sugars compared to those between sweet taste liking and the consumption of total sugars. The differences between free sugar intakes and total sugar intakes in this analysis may also suggest a distinction between these intakes, where measures of free sugar intakes may reflect a more discretionary selection of sweet-tasting high-sugar foods, while measures of total sugar intakes likely do not. Lim et al. [19] also investigated discretionary food choices, and found a greater consumption of sweet-tasting discretionary food items in sweet likers compared to sweet dislikers, although effects were not significant. The clinical relevance of any effect also requires consideration.

This analysis differs from previous studies primarily through the inclusion of a taste test composed of familiar commercially available foods, alongside the more commonly used sucrose solutions, to assess sweet taste liking. The taste test used a range of foods of different ingredients, flavours, and textures, as well as differing levels of sweet taste, and while the use of commercially available foods may have increased the ecological validity of the taste test [7], these additional characteristics may have also affected liking ratings [10,11]. Assessment of controlled levels of the tastant of interest, as used in sensory perception tests based on several concentrations of sucrose in the same food vehicle [e.g., [8,41,42]] is clearly a purer measure of liking for this specific taste. Unlike in other studies, however, our correlations between sweet taste intensity perception and sweet taste liking in these foods were based on actual perception of sweet taste intensity rather than the sucrose concentrations used to create the stimuli. In all taste assessment measures, we also chose to use continuous data rather than to categorize individuals in order to increase sensitivity and avoid false classifications [20]. The analysis is limited through our use of food diaries for the measures of free sugar and total sugar intakes, as self-report dietary assessments can be subject to both intentional and unintentional inaccuracies [22,23]. Strategies were employed to improve accuracy (training and checking by a trained researcher), and three-day food diaries, such as those used here, have been suggested as an appropriate method for assessing short-term dietary intake in free-living situations [22,23,24]. Participants also completed the diaries prior to entry into the trial and thus had not yet completed additional measures specifically on sweet taste. These analyses are limited through our use of secondary data; alternative methods of dietary assessment may have been more suitable as measures of habitual intake [22,23], but additional considerations for our primary research purpose rendered these methods less suitable for our primary research study [21]. Similarly, our sample size was limited by requirements for our primary research study [21], while a larger sample would have added value to the current investigation. Our findings are also specific to UK consumers who were consuming >5% TEI from free sugars at the time. However, the inclusion of consumers from other populations, and who are consuming <5% TEI from free sugars, would likely increase individual differences rather than reduce them.

## 5. Conclusions

Findings demonstrate some agreement between different measures of sweet taste liking and wide differences between individuals in this liking. There is also some suggestion that higher liking for sweet foods is associated with increased sweet food selection and sugar consumption in a restricted scenario, but there is very little support for the suggestion that sweet taste liking is associated with either free sugar or total sugar intakes in a free-living situation. These findings cast doubt on the assumptions that sweet taste preferences are naturally high for all and that free sugar intakes are a result of sweet taste preferences.

## Figures and Tables

**Table 1:** Descriptive statistics (mean, standard deviation (SD), and range) for sweet taste perceptions, sweet food consumption, and sugar intakes in the study sample (n = 179).

		Mean (SD)	Range
**Sweet taste perceptions**	Pleasantness for sweet foods (mm VAS (0–100))	58 (18)	2–95
	Desire to eat for sweet foods (mm VAS (0–100))	48 (22)	0–92
	Sweet taste intensity for sweet foods (mm VAS (0–100))	68 (15)	10–95
	Liking for a high sweet taste intensity (mm VAS (0–100))	50 (25)	0–99
	Pleasantness for non-sweet foods (mm VAS (0–100))	51 (16)	0–89
	Desire to eat for non-sweet foods (mm VAS (0–100))	40 (17)	0–88
	Sweet taste intensity for non-sweet foods (mm VAS (0–100))	30 (15)	0–72
**Sweet food consumption**	Weight of sweet foods consumed at breakfast, meal %weight sweet (%gram total)	25.4 (30.8)	0–100
	Energy from sweet foods consumed at breakfast, meal %energy sweet (%kcal total)	29.3 (32.5)	0–100
**Sugar consumption**	Free sugar consumption calculated from diaries, diary %FS (%TEI)	10.4 (4.9)	2.4–31.9
	Total sugar consumption calculated from diaries, diary %TS (%TEI)	16.7 (5.0)	6.3–36.4
	High-sugar-food consumption calculated from diaries, diary %HS (%gram total)	4.1 (6.7)	0–56.4
	Medium-sugar-food consumption calculated from diaries, diary %MS (%gram total)	18.0 (14.4)	0–63.7
	Sugars consumed at breakfast, meal sugars (grams)	16.8 (17.6)	0–125.2
	Energy consumed from sugars at breakfast, meal %energy sugars (%kcal total)	20.5 (17.7)	0–89.4
	Free sugars consumed at breakfast, meal free sugars (grams)	9.6 (12.7)	0–81.2
	Energy consumed from free sugars at breakfast, meal %energy free sugars (%kcal total)	11.2 (16.4)	0–89.4
**Attitudes**	SSSF: Personal Impact (1–5)	2.7 (0.6)	1.2–4.2
	SSSF: Personal Management (1–5)	3.2 (0.5)	1.8–4.5
	SSSF: Apathy (1–5)	3.4 (0.6)	2.0–4.8
	SSSF: Negativity (1–5)	3.0 (0.5)	1.7–4.1
	SSSF: Perceived Understanding (1–5)	2.9 (0.6)	1.3–4.5
	SSSF: Perceived Nonautonomy (1–5)	2.7 (0.7)	1.0–5.0
	TFEQ: Cognitive Restraint (0–100)	53 (12)	22–83
	TFEQ: Uncontrolled Eating (0–100)	52 (15)	15–85
	TFEQ: Emotional Eating (0–100)	52 (31)	0–100
**Appetite**	Hunger (mm VAS (0–100))	42 (21)	0–87
	Desire to Eat (mm VAS (0–100))	44 (23)	0–94
	Fullness (mm VAS (0–100))	31 (21)	0–99
	Prospective consumption (mm VAS (0–100))	53 (18)	8–100

**Table 2:** Regression results for regression models for meal measures of %weight consumed from sweet foods and %energy consumed from sweet foods (n = 179).

	Meal %Weight Sweet		Meal %Energy Sweet	
	R^2^ = 0.16, adj. R^2^ = 0.07, F(17,178) = 1.81, *p* = 0.03		R^2^ = 0.13, adj. R^2^ = 0.04, F(17,178) = 1.38, *p* = 0.16	
	Beta	*p*	Beta	*p*
Sweet foods pleasantness (0–100)	**0.328**	**<0.01**	0.196	0.07
Sweet foods sweet taste intensity (0–100)	−0.105	0.29	−0.053	0.60
Sweet taste intensity—pleasantness correlation (−1.0–1.0)	0.028	0.77	0.152	0.13
Liking for a high sweet taste intensity (0–100)	−0.030	0.71	−0.037	0.66
Gender (male = 1, female = 2)	−0.014	0.87	0.021	0.80
Age (years)	−0.090	0.29	0.033	0.70
BMI (kg/m^2^)	−0.108	0.20	−0.017	0.84
SSSF attitudes: Personal Impact	0.011	0.91	0.053	0.56
SSSF attitudes: Personal Management	0.119	0.15	0.097	0.25
SSSF attitudes: Apathy	−0.024	0.76	0.039	0.62
SSSF attitudes: Negativity	−0.032	0.70	0.003	0.98
SSSF attitudes: Perceived Understanding	−0.057	0.45	−0.050	0.51
SSSF attitudes: Perceived Nonautonomy	−0.037	0.63	−0.034	0.67
TFEQ: Cognitive Restraint	−0.108	0.16	−0.093	0.24
TFEQ: Uncontrolled Eating	−0.023	0.81	−0.085	0.38
TFEQ: Emotional Eating	−0.033	0.75	0.079	0.46
Hunger (0–100)	0.082	0.30	0.148	0.07

Significant effects (*p* < 0.05) are given in bold.

**Table 3:** Regression results for regression models for diary measures of %FS, %TS, meal sugars (grams), and meal free sugars (grams) in the test meal (n = 179).

	Diary %FS		Diary %TS		Meal Sugars		Meal Free Sugars		Diary %FS		Diary %TS	
	R^2^ = 0.16, adj. R^2^ = 0.07, F(17,178) = 1.78, *p =* 0.04		R^2^ = 0.19, adj. R^2^ = 0.11, F(17,178) = 2.25, *p* < 0.01		R^2^ = 0.18, adj. R^2^ = 0.09, F(17,178) = 2.03, *p =* 0.01		R^2^ = 0.17, adj. R^2^ = 0.08, F(17,178) = 1.89, *p =* 0.02		R^2^= 0.19, adj. R^2^ = 0.09, F(20,178) = 1.90, *p =* 0.02		R^2^ = 0.20, adj. R^2^ = 0.10, F(20,178) = 1.94, *p =* 0.01	
	Beta	*p*	Beta	*p*	Beta	*p*	Beta	*p*	Beta	*p*	Beta	*p*
Sweet foods pleasantness (0–100)	0.136	0.20	0.110	0.29	0.194	0.07	**0.216**	**0.04**	0.077	0.48	0.100	0.36
Sweet foods sweet taste intensity (0–100)	−0.131	0.19	−0.031	0.75	**−0.199**	**0.04**	−0.177	0.07	−0.111	0.26	−0.019	0.85
Sweet taste intensity—pleasantness correlation (−1.0–1.0)	−0.073	0.45	−0.025	0.80	0.008	0.93	0.013	0.89	−0.075	0.44	−0.020	0.84
Liking for a high sweet taste intensity (0–100)	0.098	0.23	0.051	0.53	0.034	0.67	0.035	0.67	0.107	0.19	0.054	0.51
Gender (male = 1, female = 2)	0.042	0.60	−0.107	0.18	−0.119	0.14	−0.101	0.22	0.041	0.62	−0.106	0.20
Age (years)	−0.085	0.31	**0.270**	**<0.01**	−0.030	0.72	−0.087	0.30	−0.068	0.42	**0.272**	**<0.01**
BMI (kg/m^2^)	−0.135	0.11	−0.139	0.09	0.119	0.15	0.024	0.78	−0.102	0.23	−0.127	0.14
SSSF attitudes: Personal Impact	−0.052	0.56	0.060	0.50	−0.031	0.73	−0.052	0.56	−0.054	0.55	0.062	0.48
SSSF attitudes: Personal Management	0.151	0.07	**0.218**	**<0.01**	−0.073	0.37	−0.088	0.29	0.131	0.12	**0.224**	**<0.01**
SSSF attitudes: Apathy	**−0.187**	**0.02**	−0.096	0.21	0.017	0.82	−0.035	0.65	**−0.180**	**0.02**	−0.093	0.22
SSSF attitudes: Negativity	0.028	0.74	−0.013	0.88	**0.215**	**0.01**	**0.189**	**0.02**	0.037	0.66	−0.020	0.81
SSSF attitudes: Perceived Understanding	0.046	0.54	0.026	0.72	0.014	0.85	−0.031	0.68	0.051	0.49	0.019	0.80
SSSF attitudes: Perceived Nonautonomy	0.084	0.28	−0.045	0.55	0.001	0.99	0.041	0.59	0.092	0.23	−0.043	0.57
TFEQ: Cognitive Restraint	**−0.193**	**0.01**	−0.059	0.44	−0.047	0.54	−0.054	0.48	**−0.171**	**0.03**	−0.053	0.49
TFEQ: Uncontrolled Eating	0.025	0.79	−0.115	0.22	−0.137	0.15	−0.114	0.23	0.019	0.84	−0.122	0.20
TFEQ: Emotional Eating	−0.006	0.95	−0.152	0.14	0.079	0.45	0.013	0.90	0.014	0.90	−0.137	0.19
Hunger (0–100)	−0.096	0.22	0.072	0.35	0.146	0.06	0.105	0.18	−0.111	0.16	0.063	0.42
Meal %weight sweet									0.186	0.06	0.006	0.95
Meal sugars									−0.042	0.65	−0.001	0.99
Meal %energy sugars									0.054	0.57	0.075	0.43

Significant effects (*p* < 0.05) are given in bold.

## Data Availability

The raw data supporting the conclusions of this article will be made available by the authors on request.

## Supplementary Materials

The following supporting information can be downloaded at: https://www.mdpi.com/article/10.3390/nu16213672/s1, Figure S1: Mean pleasantness rating for sweet foods across the population (n = 179); Figure S2: Mean desire to eat rating for sweet foods across the population (n = 179); Figure S3: Liking for a 1M sucrose solution across the population (n = 179); Table S1: Foods provided in the buffet-style breakfast meal; Table S2: Full characteristics of the study sample (n = 179); Table S3: Results of the regression analyses investigating the relationship between liking for sweet and non-sweet foods and liking for a high sweet taste intensity, while also considering hunger (n = 179).
