# Supplementary material for: Liking for Sweet Taste, Sweet Food Intakes, and Sugar Intakes
Source: Nutrients. 2024 Oct 29;16(21):3672. doi: 10.3390/nu16213672 (PMC11547215; doi:10.3390/nu16213672)
Supplement: Supplementary file 1 [file nutrients-16-03672-s001.zip › nutrients-3233581-supplementary.pdf]

**Liking for sweet taste, sweet food intakes, and sugar intakes**
Katherine M Appleton

## SUPPLEMENTARY MATERIALS

**Table S1:** Foods provided in the buffet-style breakfast meal

| Food | Taste | Amount provided (items) |
|---|---|---|
| Plain bagels (New York Bakery Co.), (Grupo Bimbo UK Ltd, Rotherham, UK) | Non-sweet | 6 half bagels |
| Cinnamon & Raisin bagels (New York Bakery Co.), (Grupo Bimbo UK Ltd, Rotherham, UK) | Sweet | 6 half bagels |
| Butter (Lurpak spreadable) (Arla Foods Ltd., Leeds, UK)[1] | Non-sweet | 1 x 250g tub[2] |
| Strawberry Jam (Hartley's) (Hain Celestial, Leeds, UK | Sweet | 1 x 300g jar[2] |
| Honey (Rowse, runny honey) (Rowse Honey, Ltd., Oxfordshire, UK) | Sweet | 1 x 454g jar[2] |
| Cream Cheese (Philadelphia) (Mondelez UK, Uxbridge, UK) | Non-sweet | 1 x 165g tub[2] |
| Peanut butter (Whole Earth, smooth) (Kallo Foods Ltd., Surrey, UK) | Non-sweet | 1 x 454g jar[2] |
| Cornflakes (Kellogg's) (Kellogg Marketing & Sales Company (UK) Ltd., UK) | Non-sweet | 1 x 500g box[2] |
| Crunchy Nut Cornflakes (Kellogg's) (Kellogg Marketing & Sales Company (UK) Ltd., UK) | Sweet | 1 x 500g box[2] |
| Branflakes (Kellogg's) (Kellogg Marketing & Sales Company (UK) Ltd., UK) | Non-sweet | 1 x 500g box[2] |
| Fruit 'n Fibre (Kellogg's) (Kellogg Marketing & Sales Company (UK) Ltd., UK) | Sweet | 1 x 500g box[2] |
| Semi-skimmed milk (Cravendale) (Arla Foods Ltd., Leeds, UK)[1] | Non-sweet | 1 litre |
| Pure Orange Juice (Sainsbury's Supermarkets Ltd., London, UK) | Sweet | ½ litre |
| Tea (Yorkshire Tea teabags) (Taylors of Harrogate, North Yorkshire, UK) | Non-sweet | 160 teabags[2] |
| Decaffeinated tea (Yorkshire Tea teabags) (Taylors of Harrogate, North Yorkshire, UK) | Non-sweet | 160 teabags[2] |
| Coffee (Nescafe instant coffee powder) (Nestle UK, York, UK) | Non-sweet | 1 x 200g jar[2] |
| Decaffeinated coffee (Nescafe instant coffee powder) (Nestle UK, York, UK) | Non-sweet | 1 x 200g jar[2] |
| Sugar (granulated) (Tate & Lyle Sugars, Nottinghamshire, UK) | Sweet | 200g |
| Sweetener (sucralose-based powder) (Tate & Lyle Sugars, London, UK) | Sweet | 75g |
| Water (tap, non-sparkling) | Non-sweet | 1 litre |

[1]Plant-based alternatives for select dairy products were provided where requested.
[2]Amount provided ranged from half to an almost full pack

**Table S2:** Full characteristics of the study sample (n = 179)

| Characteristic | Detail | Number |
|---|---|---|
| Gender | Male | 21 |
| | Female | 158 |
| Age (years) | 40.6 (SD = 13.7) | |
| BMI ($kg/m^2$) | 27.8 (SD = 5.89) | |
| Ethnicity | White British | 154 |
| | White non-British | 16 |
| | Other | 9 |
| Occupation | Unemployed | 18 |
| | Routine / semi-routine / lower supervisory | 40 |
| | Intermediate / lower managerial | 94 |
| | Higher managerial | 27 |
| Education | Other / GCSE | 16 |
| | A-level / Inter-Baccalaureate or equivalent | 55 |
| | Undergraduate degree | 66 |
| | Postgraduate degree | 42 |
| Income | Very insufficient | 2 |
| | Insufficient | 20 |
| | Sufficient | 157 |
| Main cook | Yes | 138 |
| | No | 41 |
| Diet type | Omnivore | 146 |
| | Vegetarian | 24 |
| | Vegan | 2 |
| | Other | 7 |
| Religion | Likely to affect me | 1 |
| | Unlikely to affect me | 178 |

**Figure S1:** Mean pleasantness rating for sweet foods across the population *(*n = 179)

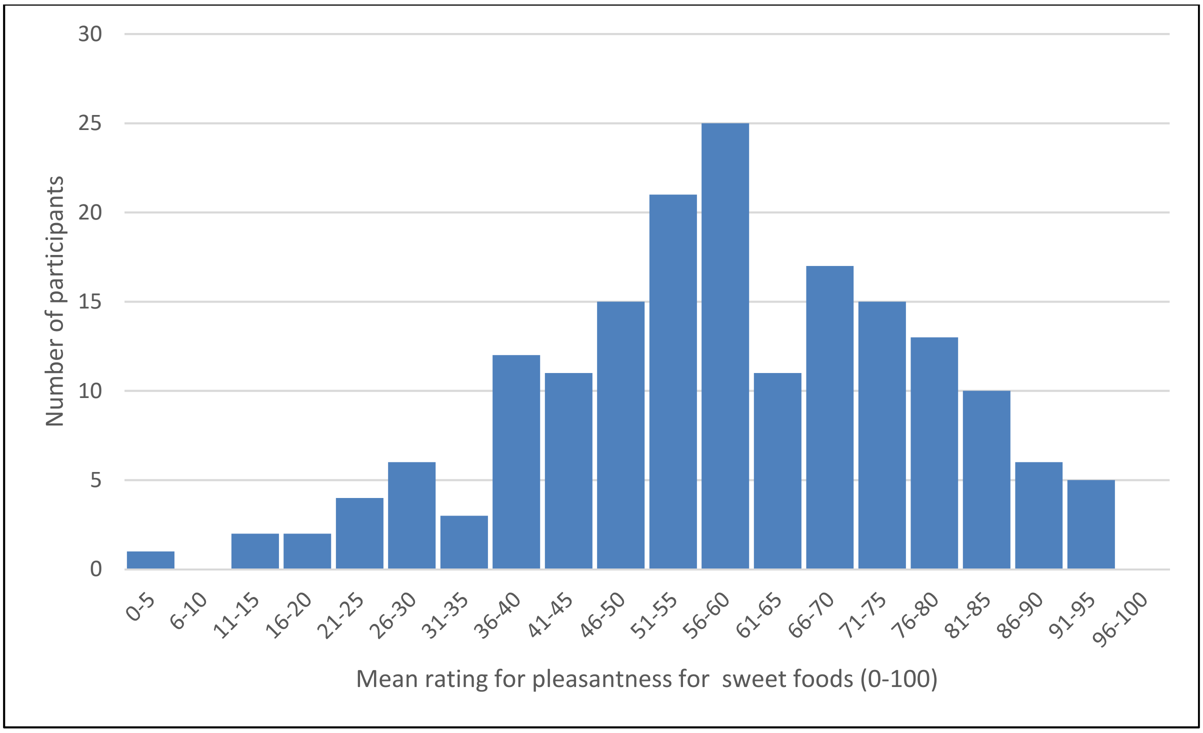


**Figure S2:** Mean desire to eat rating for sweet foods across the population (n = 179)

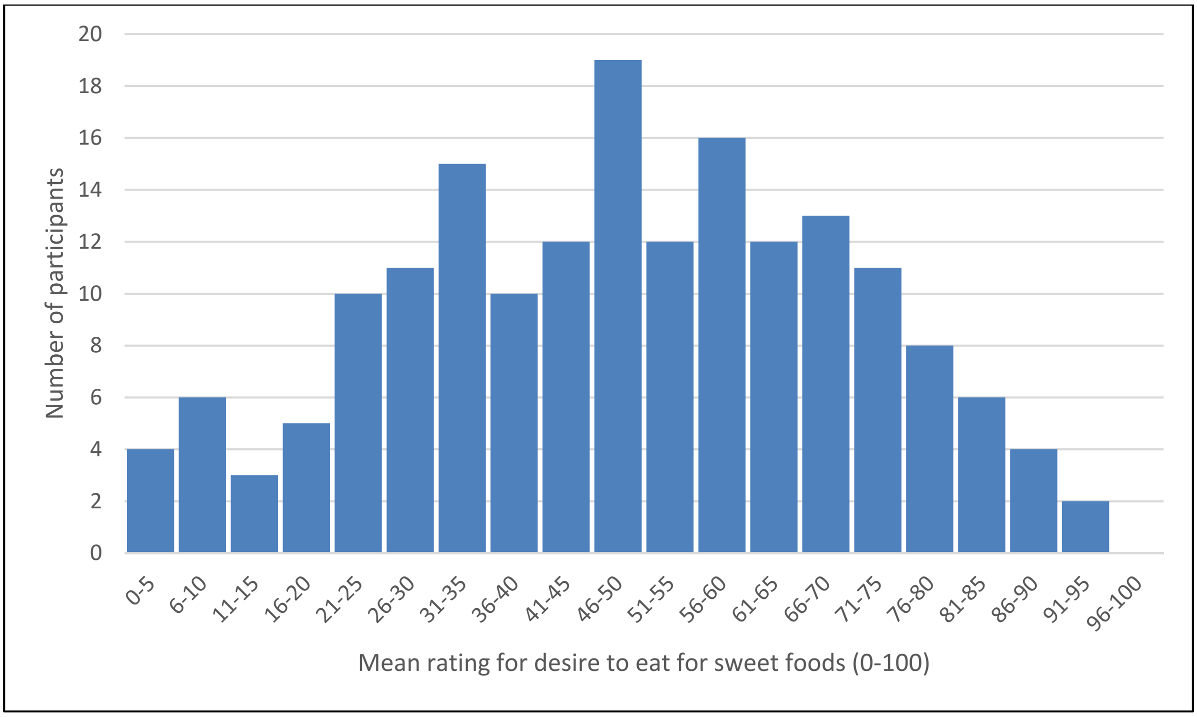

**Figure S3:** Liking for a 1M sucrose solution across the population (n = 179)

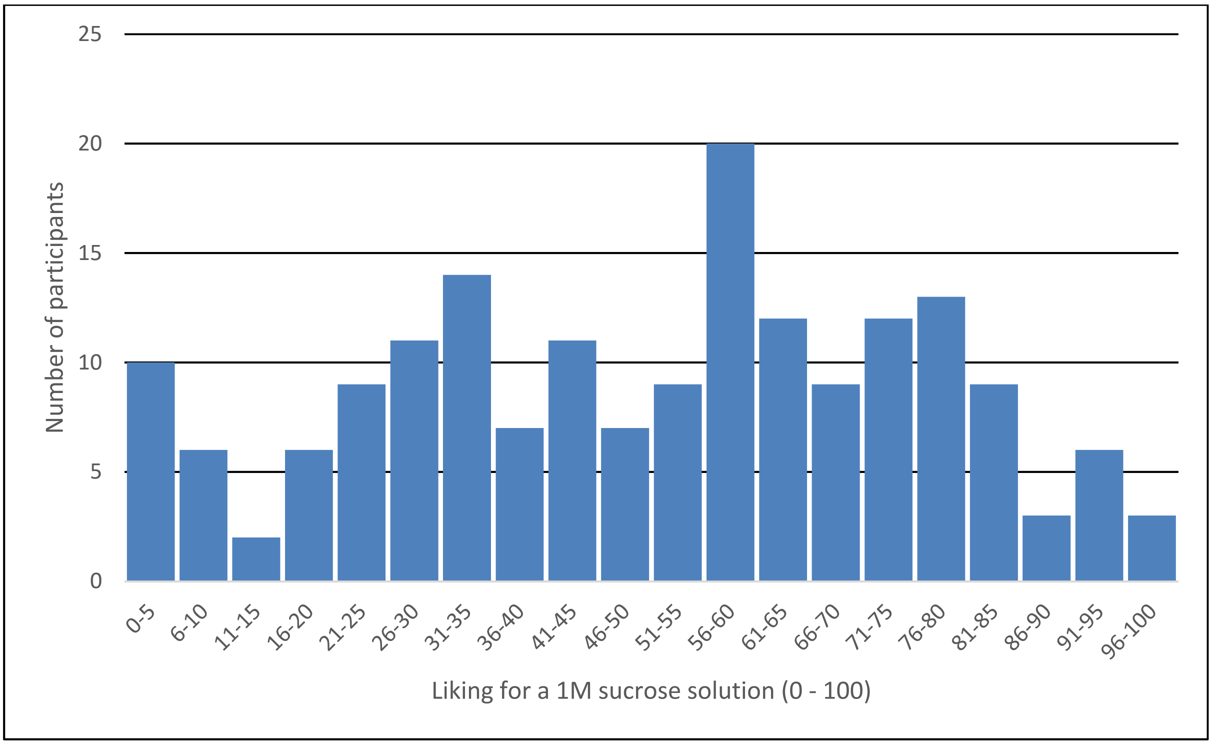


**Table S3:** Results of the regression analyses investigating the relationship between liking for sweet and non-sweet foods and liking for a high sweet taste intensity, while also considering hunger (n = 179)

| | R2 | adj. R2 | F(2,178) | p(F) | High sweet taste intensity liking Beta | p(Beta) |
|---|---|---|---|---|---|---|
| Pleasantness: sweet foods | .09 | .08 | 8.91 | < .01 | .293 | < .01 |
| Desire to eat: sweet foods | .11 | .10 | 10.90 | < .01 | .303 | <.01 |
| Pleasantness: non-sweet foods | .01 | .00 | 0.74 | .48 | .012 | .87 |
| Desire to eat: non-sweet foods | .09 | .08 | 8.27 | < .01 | .121<br>Hunger = .254 | .10<br><.01 |
